# Genotyping of *BCL11A* and *HBS1L-MYB* SNPs associated with fetal haemoglobin levels: a SNaPshot minisequencing approach

**DOI:** 10.1186/1471-2164-15-108

**Published:** 2014-02-06

**Authors:** Pavlos Fanis, Ioanna Kousiappa, Marios Phylactides, Marina Kleanthous

**Affiliations:** 1 Molecular Genetics Thalassaemia Department, The Cyprus Institute of Neurology and Genetics, 6 International Airport Avenue, Agios Dometios, Nicosia 1683, Cyprus

**Keywords:** BCL11A, HBS1L-MYB, HbF, Thalassaemia, SCD, SNaPshot minisequencing, Multiplex PCR, Polymorphisms

## Abstract

**Background:**

B-thalassaemia and sickle cell disease (SCD) are two of the most common monogenic diseases that are found in many populations worldwide. In both disorders the clinical severity is highly variable, with the persistence of fetal haemoglobin (HbF) being one of the major ameliorating factors. HbF levels are affected by, amongst other factors, single nucleotide polymorphisms (SNPs) at the *BCL11A* gene and the *HBS1L-MYB* intergenic region, which are located outside the *β-globin locus*. For this reason, we developed two multiplex assays that allow the genotyping of SNPs at these two genomic regions which have been shown to be associated with variable HbF levels in different populations.

**Results:**

Two multiplex assays based on the SNaPshot minisequencing approach were developed. The two assays can be used to simultaneous genotype twelve SNPs at the *BCL11A* gene and sixteen SNPs at *HBS1L-MYB* intergenic region which were shown to modify HbF levels. The different genotypes can be determined based on the position and the fluorescent colour of the peaks in a single electropherogram. DNA sequencing and restriction fragment length polymorphism (PCR-RFLP) assays were used to verify genotyping results obtained by SNaPshot minisequencing.

**Conclusions:**

In summary, we propose two multiplex assays based on the SNaPshot minisequencing approach for the simultaneous identification of SNPs located at the *BCL11A* gene and *HBS1L-MYB* intergenic region which have an effect on HbF levels. The assays can be easily applied for accurate, time and cost efficient genotyping of the selected SNPs in various populations.

## Background

B-thalassaemia and SCD are common autosomally inherited disorders that are found in many populations around the world. B-thalassaemia is caused by mutations that lead to low or complete absence of expression of the β-globin gene while in SCD a point mutation in the β-globin gene causes changes in the protein structure [1,2]. In β-thalassaemia the excess of unbound alpha globin chains precipitate in the red cell precursors leading to their destruction in the bone marrow resulting in ineffective erythropoiesis. Similarly, excess of unbound alpha globin chains induce membrane damage in mature thalassaemic red cells leading to haemolysis [3]. The pathogenesis of SCD initiates when the deoxy-sickle haemoglobin molecules are polymerized leading to the deformation of the structure of the red blood cell. In both disorders, the clinical phenotypes are highly variable, ranging from relatively mild to severe anaemia, due to environmental and genetic factors [2,4].

One of the major ameliorating factors of clinical severity, in both conditions, is the persistence of HbF (α_2_γ_2_) production [5]. During fetal life, HbF is the predominant haemoglobin produced which switches to adult haemoglobin (HbA, α_2_β_2_) around birth [6]. By the end of the first year of life the levels of HbF decrease to less than 1% of total haemoglobin. In some individuals haemoglobin switching is not complete and a significant amount of HbF is produced during adulthood, resulting in a condition called hereditary persistence of fetal haemoglobin (HPFH) [7]. In SCD and β-thalassaemia, high γ-globin expression inhibits HbS polymerization and α-globin precipitation, respectively [8]. Point mutations at the promoter of the γ-globin gene and deletions within the β-globin gene cluster can result in increased levels of HbF [2]. However, HbF levels are also affected by genetic factors outside of the β-globin gene cluster.

Genetic association studies have identified SNPs in major loci that are associated with the variation of HbF levels in patients with SCD or β-thalassaemia and in healthy adults. These loci are the *BCL11A* gene on chromosome 2 (2p16.1) and the *HBS1L-MYB* intergenic region on chromosome 6 (6q23.3) [9,10]. The polymorphisms of *BCL11A* gene that have been described and are associated with variable HbF levels are located within the second intron of this gene. The *HBS1L-MYB* intergenic polymorphisms (HMIP) are present in three linkage disequilibrium (LD) blocks with most of the effect on HbF levels and numbers of F cells contributed by the second block [9-13].

The *BCL11A* gene encodes a zinc finger transcription factor that represses HbF synthesis (γ-globin expression) [14-16]. Expression of the two major isoforms (XL, L) of BCL11A protein in human adult erythroid cells is needed to repress the *γ-globin* expression [14,15]. The BCL11A protein occupies sites within the locus control region (LCR) and intergenic regions of the *β-globin* locus but does not associate with the promoter of the *γ-globin* genes indicating that the regulation of *γ-globin* expression is a complex process [17]. Moreover, BCL11A interacts with transcription factors such as GATA1, FOG1 and SOX6 and is required for the recruitment of the NuRD and LSD1/CoREST chromatin remodeling complexes to the *β-globin* locus in primary erythroid cells [14,16,18]. *MYB* is a proto-oncogene that is encoding the c-MYB transcription factor, which plays an essential role in erythroid differentiation [19-21]. Absence of c-myb in mice is lethal due to failure to develop haematopoiesis in fetal liver [19]. Disruption of the *Hbs1l-Myb* intergenic region in mice suppresses Myb levels and leads to elevated expression of embryonic globins and haematopoietic parameters similarly to human HPFH [22]. Moreover, down-regulation of MYB transcription factor through over-expression of microRNAs miR-15a and 16-1 results in elevations in HbF gene expression [23].

Thus, analysis of SNPs that are located in the *BCL11A* gene and the *HBS1L-MYB* intergenic region and have an effect on HbF levels can provide crucial genetic information enabling patient stratification and can help predict the severity of disease in newborns with β-thalassaemia and SCD. Furthermore, in the future such information could help clinicians to provide more personalized patient care according to the expected phenotype. Finally, determination of these SNPs will help to better understand the molecular mechanisms of action of modifying factors influencing the production of haemoglobin.

Routinely, identification of SNPs is based on detection by amplification-refractory mutation system (ARMS), PCR followed by PCR-RFLP and Sanger DNA sequencing. These assays are costly and time consuming in cases where several SNPs need to be studied, hence there is a need for an accurate, sensitive and low cost assay for detection of multiple polymorphisms in a single assay.

Here we report the development of two multiplex assays based on the SNaPshot-minisequencing approach for simultaneous screening of twelve and sixteen known SNPs, which have an effect on HbF levels in different populations, in the *BCL11A* gene and *HBS1L-MYB* intergenic region, respectively. In each assay we use multiplex PCR followed by multiplex single-base extension with a fluorescent dideoxynucleotide of a primer which anneals immediately adjacent to the SNP under investigation. The extended products are separated and fluorescently detected by capillary electrophoresis. This molecular tool is rapid and cost effective for the detection of multiple SNPs in the *BCL11A* gene and *HBS1L-MYB* intergenic region. It can help associate specific SNPs and/or haplotypes to the variation of HbF levels in particular populations but also to enhance our general understanding of their contribution to HbF modulation.

## Methods

### Biological samples

We used genomic DNA samples from one individual with high levels of HbF, and 25 anonymous confirmed β-thalassaemia patients previously genotyped for haemoglobinopathy mutations in the Molecular Genetics Thalassaemia Department, The Cyprus Institute of Neurology and Genetics. The study was conducted in accordance with the Declaration of Helsinki and was approved by the National Bioethics Committee of Cyprus (Reference number: ΕΕΒΚ/ΕΠ/2012/05). The genomic DNA was extracted from peripheral blood using the Gentra Puregene Kit (Qiagen) according to the manufacturer’s protocol and kept at -80°C for long-term storage. The DNA concentration and purity were measured using the Nanodrop ND-1000 spectrophotometer (NanoDrop Technologies).

### SNP selection

A set of twelve *BCL11A* and sixteen *HBS1L-MYB* SNPs were chosen based on various published studies [9-13,24-33] and the new updated haemoglobinopathy sequence variation database, the IthaGenes of the ITHANET Portal at http://www.ithanet.eu/db/ithagenes. Taken together, the data suggest that these polymorphisms are associated with variable HbF levels in different ethnic populations with HPFH, β-thalassaemia, sickle cell anaemia, β-thalassaemia heterozygotes and HbE heterozygotes. The selected SNPs are listed in Table 1, where the reference SNP number is indicated, along with their HGVS name and the geographical/ethnic origin of each SNP study cohort.

**Table 1:** **Selected *BCL11A *** **and *HBS1L-MYB *** **intergenic region SNPs**

**Ref SNP**	**HGVS name**^***a***^	**Origin of population (Cohort)**
***BCL11A***		
rs11886868	NC_000002.11:g.60720246C>T	African Americans SCD (Cooperative Study of Sickle Cell Disease (CSSCD)) [12], Brazil SCD [12], Sardinians [24], French β-thalassaemia [25]
rs4671393	NC_000002.11:g.60720951A>G	African Americans SCD (CSSCD) [11,12], Brazil SCD [12], North Europeans [10], Chinese β-thalassaemia [26]
rs7557939	NC_000002.11:g.60721347G>A	African Americans SCD (CSSCD) [12], Brazil SCD [12]
rs6732518	NC_000002.11:g.60708597C>T	North Europeans [10], African Americans SCD (CSSCD) [27], Africans SCD (Silent Infarct Transfusion (SIT) Trial) [28], Thai β-thalassaemia heterozygotes or HbE heterozygotes [27], African Americans SCD [29], Hong Kong β-thalassaemia heterozygotes [27]
rs10189857	NC_000002.11:g.60713235A>G	African Americans SCD (CSSCD) [11], Sardinians β^0^-thalassaemia [30]
rs6545816	NC_000002.11:g.60714861A>C	Thai and Thai-Chinese β^0^-thalassaemia/HbE [31], Thai β-thalassaemia heterozygotes or HbE heterozygote [29]
rs7599488	NC_000002.11:g.60718347C>T	African Americans SCD (CSSCD) [11]
rs1427407	NC_000002.11:g.60718043T>G	North Europeans [10], Sardinians β^0^-thalassaemia [30]
rs766432	NC_000002.11:g.60719970C>A	North Europeans [10], Chinese (Hong Kong) β-thalassaemia heterozygotes [29,32], Thai and Thai-Chinese β^0^-thalassaemia/HbE [31], Chinese β-thalassaemia [26], Thai β-thalassaemia heterozygotes or HbE heterozygotes [27], Hong Kong β-thalassaemia heterozygotes [27], African Americans SCD (CSSCD) [27], Africans SCD (SIT Trial) [28], African Americans SCD [29]
rs10184550	NC_000002.11:g.60729294G>A	African Americans SCD [29]
rs7606173	NC_000002.11:g.60725451G>C	Africans SCD (SIT Trial) [28]
rs6706648	NC_000002.11:g.60722040C>T	Africans SCD (SIT Trial) [28]
***HBS1L-MYB***		
rs28384513	NC_000006.11:g.135376209T>G	African Americans SCD (CSSCD) [11,12], Brazil SCD [12]
rs7776054	NC_000006.11:g.135418916A>G	African Americans SCD (CSSCD) [12], Brazil SCD [12]
rs9399137	NC_000006.11:g.135419018T>C	African Americans SCD (CSSCD) [12], Brazil SCD [12], North Europeans [9,10,33], Sardinians β^0^-thalassaemia [30],Thai and Thai-Chinese β^0^-thalassaemia/HbE [31], French β-thalassaemia [25], Chinese (Hong Kong) β-thalassaemia heterozygotes [32], Africans SCD (SIT Trial) [28]
rs9389268	NC_000006.11:g.135419631A>G	African Americans SCD (CSSCD) [12], Brazil SCD [12]
rs4895441	NC_000006.11:g.135426573A>G	African Americans SCD (CSSCD) [12], Brazil SCD [12], Sardinians [24], Sardinians β^0^-thalassaemia [30], Thai and Thai-Chinese β^0^-thalassaemia/HbE [31], North Europeans [9], Chinese β-thalassaemia [26], Africans SCD (SIT Trial) [28], Chinese β-thalassaemia heterozygotes [13]
rs6929404	NC_000006.11:g.135454027C>A	North Europeans [9,33]
rs9402686	NC_000006.11:g.135427817G>A	African Americans SCD (CSSCD) [11], North Europeans [9], Chinese β-thalassaemia [26]
rs1320963	NC_000006.11:g.135443212A>G	North Europeans [10]
rs6904897	NC_000006.11:g.135382980T>G	North Europeans [10], Sardinians β^0^-thalassaemia [30]
rs35959442	NC_000006.11:g.135424179C>G	Chinese β-thalassaemia [26]
rs9376090	NC_000006.11:g.135411228T>C	North Europeans [9], Chinese β-thalassaemia [26]
rs4895440	NC_000006.11:g.135426558A>T	North Europeans [9], Chinese β-thalassaemia [26]
rs9494142	NC_000006.11:g.135431640T>C	Chinese β-thalassaemia [26]
rs9402685	NC_000006.11:g.135419688T>C	North Europeans [9], Chinese β-thalassaemia [26]
rs11759553	NC_000006.11:g.135422296A>T	North Europeans [9], Chinese β-thalassaemia [26]
rs6934903	NC_000006.11:g.135451564T>A	Chinese β-thalassaemia heterozygotes [13]

^*a*^HGVS Name: Human Genome Variation Society reference name (Reverse direction of the SNPs sequences for *BCL11A* gene).

### Design and optimization of PCR and minisequencing primers

The genetic regions including the selected *BCL11A* and *HBS1L-MYB* SNPs were identified by the nucleotide Basic Local Alignment Search Tool (BLAST) and a total of forty-four forward and reverse PCR oligonucleotide primers were designed with the Biomaths Calculator program at http://www.promega.com/techserv/tools/biomath/calc11.htm. All oligonucleotides were designed to have melting temperatures of 57-64°C and their lengths ranged between 17 and 29 nucleotides (nt). The primer sequences were screened to avoid homology to other loci or repetitive sequences in the genome by using the BLAST alignment tool. Specifically, the primer sequences were screened using the “Human genomic plus transcript (Human G + T)” database, the “megablast” algorithm and were checked for low complexity regions. It is highly recommended, when looking for homologies, to use the BLAST tool, which is more sensitive rather than other alignment tools such as BLAT [34]. When using the BLAST tool for primer screening, appropriate care should be taken for the presence of pseudogenes or duplicated genes that could interfere with the assay. Secondary structures and potential primer-primer interactions were predicted using the OligoAnalyzer 3.1 tool at http://eu.idtdna.com/analyzer/Applications/OligoAnalyzer/. All multiplex PCR primer sequences are listed in Table 2, along with three sequencing primers that were designed in a similar way. For each region (*BCL11A* or *HBS1L-MYB*), the amplicons encompassing the SNPs of interest were amplified in two separate multiplex PCR (mPCR) reactions, A and B. The sizes of the amplicons ranged from 69 to 649 bp for the *BCL11A* mPCRs and from 60 to 221 bp for the *HBS1L-MYB* mPCRs.

**Table 2:** ***BCL11A *** **and *HBS1L-MYB *** **multiplex PCR amplification primers**

**SNP**	**Multiplex PCR primers (5′-3′)**		**mPCR Reaction**	**Amplicon fragment length (bp)**	**Concentration (μM)**
	**Forward**	**Reverse**			
***BCL11A***					
rs11886868, rs766432	CACACCATGGATGAATCCCAGA^α,*^	TGGTGCTACCCTGAAAGACGG^α,*^	B	441	0.1
rs4671393, rs7557939	CCTTGTTTACAGAGGGGCAACC^α,*^	GCCTTGGGAAGAAAGACAGCAT^α,*^	B	649	0.4
rs6732518	TGGGTGACCCTCTGACTCCT^*^	GCTTTAACGCACTACACCCCAC^α,*^	A	69	0.1
rs10189857	AGCCATGGTCCACCCACAGA^α,*^	TTCCAGGTCCCCCAGAAGTAGC^*^	B	411	0.1
rs6545816	GGTTGATGAGAAAATTACCGCATTA^α,*^	AACGAGGGTGTTCAAAGTAACCA^*^	A	103	0.3
rs7599488, rs1427407	CTGGCCGGGAGCATTTCAA^α,*^	TTTAACCTTCTTAGCACCCACAAA^*^ GCACAGCATGTGACATGATATTC^b^	B	515	0.6
					0.5
rs10184550	CCTGATCTCTGATTGTTGCTTTGA^*^	TGTACCACCAGAAGTCCTGGAAA^α,*^	A	120	0.4
rs7606173	ACACCCTGTGATCTTGTGG^α,*^	GCCAACAGTGATAACCAGC^*^	A	201	0.8
rs6706648	GAAGCTTCCCCTGTCTGCA^α,*^	TGAGTGCGTATTTGTAAAGTTCC^*^	A	333	0.6
***HBS1L-MYB***					
rs28384513	CCTTGAGCTACCTACGCCAG^*^	CTTTCTCAGATTATCAGGAACCAAATTT^*^ GCCCACTGTGTGCTTAATGAAA^b^	A	66	0.4
					0.5
rs7776054, rs9399137	ATATGCAATATTTGTAATTTGTGTTCTGC^α,*^	TTAACTATATCTGTGCACAGAAATACAG^α,*^	B	190	0.8
rs9389268, rs9402685	TGCAACCTCCGCTTCCA^α,*^	TAGCTCACACCTGTAATCATGC^α,*^	A	221	0.2
rs4895441, rs4895440	GGAAACCAGTTTAGAAAGCGTGG^*^	TCTCTCTGGATCTCCCTGTC^α,*^	B	122	0.3
rs6929404	GCAGTGAGATTTCTATTATTAGGCTC^α,*^	CTGACTAAACTTCTAATCAAAGGCAT^*^	B	136	0.3
rs9402686	GAGCAGAGAAGTTTAAAGTGTGTG^*^	TGGACAAAACTGCCCTTTGTC^α,*^	B	150	0.4
rs1320963	ATGGCTTGGTAAGGACTTAAGAG^*^	CCTGAGAGTTCTTTTGGGATCTT^*^	B	60	0.4
	TTCAACTTTCCACTGAGTTTTCG^b^				0.5
rs6904897	ATCCAGGCTTGCCAAATATACCT^*^	GACTTGCCTGTGCTGGAAATT^α,*^	A	99	0.3
rs35959442	TGACCCAGAGCGTCCAAG^α,*^	GTTACATCTGCAGCTGACACC^*^	A	160	0.8
rs9376090	GATATGGCCAATACCATATGAAGCTAA^α,*^	AGCTGGGCCTCATTTGTTC^*^	B	81	0.2
rs9494142	AGGAAGTGTCTTTGGTCTCTCAG^α,*^	TGAGACTCCATCTCAAAAAACAACA^*^	A	143	0.4
rs11759553	TTGCCAGGCTGTCTTGCA^α,*^	GTTCTGCAGGGTCCTTTGG^*^	B	107	0.2
rs6934903	CCTTGGCCCACATCGCT^α,*^	TAGAACCTGAAACAGTACTCCACA^*^	A	130	0.2

*Used as PCR-RFLP primers at final concentration of 0.2 μM as described in the text.

^α^Used also as sequencing primers at final concentration of 0.5 μM as described in the text.

^b^Used only as sequencing primers as described in the text.

SNPs for each of the two genetic loci were genotyped in one multiplex SNaPshot minisequencing assay. Minisequencing primers were designed with their 3′ base corresponding to the base immediately before the SNP to be investigated. Therefore, for each SNP, the base subsequent to the minisequencing primer would identify the polymorphism. Minisequencing primer design was performed according to the recommendations of Sanchez *et al*., 2003 [35] using the Biomaths Calculator program. The sequences of the twenty-eight primers used in these two assays are listed in Table 3. The sequences of the minisequencing primers were checked for possible hairpin structures, primer-dimer formation or homology to other loci, as described above. In order to allow an adequate distribution of the primer-extension products, non-homologous neutral sequences were incorporated at the 5′-ends of the minisequencing primers to adjust their lengths and obtain a more balanced base composition [36]. For six primers such of an addition was not necessary while for one long primer, a poly(C)-tail was also added to the 5′-end of the neutral sequence [35]. Thus, the length of the minisequencing primers ranged from 20 to 57 nt for *BCL11A* and from 16 to 68 nt for *HBS1L-MYB.* To allow sufficient electrophoretic resolution of the multiplex extension products, minisequencing oligonucleotides that are expected to give the same fluorescent signal for SNPs in neighboring positions were designed so that they differ by a minimum of 4 nt in length. All PCR oligonucleotides were purified by standard desalting while minisequencing oligonucleotides were purified by high performance liquid chromatography (Metabion International AG).

**Table 3:** ***BCL11A *** **and *HBS1L-MYB *** **minisequencing primers**

**SNP**	**Primer direction**	**Minisequencing primers (5′-3′)**^***a***^	**Size (bp)**	**Concentration (μM)**	**Detected alleles**^***b***^
***BCL11A***					
rs11886868	F	CAGAATCATTCTGCTCTGTG	20	0.02	G/A
rs4671393	F	acaaGGAATCTTAATTTCCTGCAC	24	0.1	T/C
rs7557939	F	aaCACCCTCTCTCACTCTTG	20	0.1	C/T
rs6732518	F	aactaggtgccacgtcgtgaaagtctgacaaGACTCCTGAGAGCATGT	48	0.02	G/A
rs10189857	F	gtctgacaaGCTTGTCACAGTTCTCTAC	28	0.2	T/C
rs6545816	F	tctgacaaATTAAAATTAAGCCTCTTGCTTTT	32	0.05	T/G
rs7599488	R	aactgactaaactaggtgccacgtcgtgaaagtctgacaaTTAGTCTCAGCCACCTG	57	0.2	G/A
rs1427407	F	gtctgacaaTTCAAGTAGATATCAGAAGGGAA	32	0.1	A/C
rs766432	F	aggtgccacgtcgtgaaagtctgacaaATGAATGACTTTTGTTGTATGTAAA	52	0.05	G/T
rs10184550	F	ctgacaaTTGCTTTGATAAGTATCTATACAAATATT	36	0.05	C/T
rs7606173	F	taggtgccacgtcgtgaaagtctgacaaTGTCCTGTGAGCGGTC	44	0.2	C/G
rs6706648	F	acgtcgtgaaagtctgacaaCTTGCCTCCCCCTGAC	36	0.05	G/A
***HBS1L-MYB***					
rs28384513	F	aggtgccacgtcgtgaaagtctgacaaTACCTACGCCAGCGTTC	44	0.15	T/G
rs7776054	F	actaggtgccacgtcgtgaaagtctgacaaCAATATTTGTAATTTGTGTTCTGCTTCTAC	60	0.1	A/G
rs9399137	F	CAATAATGTAATTAACTGAACATATGGTTATT	32	0.15	T/C
rs9389268	F	GATTACAGGCGCATGCAACC	20	0.3	A/G
rs4895441	R	actgactaaactaggtgccacgtcgtgaaagtctgacaaCTTACTCAGTTCTCTGCTCATGTA	63	0.1	A/G
rs6929404	F	gtgaaagtctgacaaAAAAGTCTAGAGCACAAAAATTAAAATAA	44	0.1	C/A
rs9402686	F	GTTTAAAGTGTGTGACCTTGAGAC	24	0.05	G/A
rs1320963	R	agtctgacaaCTGAGAGTTCTTTTGGGATCTTTCAC	36	0.05	A/G
rs6904897	R	tcaggtgccacgtcgtgaaagtctgacaaGTGTATTTCTTTTGGTTGTGCATACA	55	0.1	T/G
rs35959442	R	gtctgacaaGCTCTTATAGCAGTCTACAGCAG	32	0.05	C/G
rs9376090	F	AGTCTAGCTGAGTGTTAGCC	20	0.05	T/C
rs4895440	F	GCGTGGCTGGGGAAAG	16	0.1	A/T
rs9494142	F	ccacgtcgtgaaagtctgacaaCAGTCAATTCGATTCTACTACTGACA	48	0.05	T/C
rs9402685	F	ccccccaactgactaaactaggtgccacgtcgtgaaagtctgacaaATGTTTCACCGTGTTGCTCAGG	68	0.1	T/C
rs11759553	F	aaagtctgacaaTGATTGGGGTAGGCCATAGG	32	0.05	A/T
rs6934903	F	tcaggtgccacgtcgtgaaagtctgacaaCCTGCATAAGTGTCGAATCTCTA	52	0.05	T/A

^*a*^Small letters indicate neutral sequence and poly(C) tail, and capital letters the specific target sequence.

^*b*^Detected Major/Minor alleles.

### Multiplex minisequencing detection of SNPs

Genotypic detection of *BCL11A* and *HBS1L-MYB* SNPs was performed separately for each target region using two mPCRs followed by a multiplex SNaPshot minisequencing reaction [37]. In the first step, two amplification reactions were performed (mPCRA and mPCRB) using 100 ng of genomic DNA, 1X Amplitaq Gold Buffer (Applied Biosystems), 200 mΜ each dNTP (Invitrogen), 1.25 units of Amplitaq Gold DNA Polymerase (Applied Biosystems), primer mix (final concentrations ranged between 0.1-0.8 μM in mPCRA and 0.1-0.4 μM in mPCRB for *BCL11A* and 0.2-0.8 μM in both mPCRA and mPCRB for *HBS1L-MYB*, see Table 2), and double distilled water to a final volume of 25 μl. The amplification reaction was carried out in a Veriti 96-well Thermal Cycler (Applied Biosystems). The reaction was initialized at 95°C for 10 min, followed by 35 cycles for 30 sec at 95°C, 60 sec at 61°C; 90 sec at 72°C and a final extension for 7 min at 72°C. Amplified products were analyzed in a 2% and 3% w/v ethidium bromide-stained agarose gel for *BCL11A* and *HBS1L-MYB* respectively. The sizes of the amplified PCR products were 69 to 333 bp in mPCRA and 411 to 649 bp in mPCRB for *BCL11A* and 66 to 221 bp in mPCRA and 60 to 190 bp in mPCRB for *HBS1L-MYB* (see Table 2).

In order to avoid the participation of primers and unincorporated dNTPs in the subsequent minisequencing primer extension reaction, 2.5 μl of mPCRA and 2.5 μl mPCRB products were combined and treated with an *Exo* I/Antarctic Phosphatase mix consisting of 5 units of *E. coli* exonuclease I (*Exo* I, New England Biolabs) and 1 unit of Antarctic phosphatase (New England Biolabs). The reaction mix was incubated at 37°C for 1 hour and then the enzymes were inactivated by incubating at 75°C for 15 min. The treated PCR products were used as template in a multiplex SNaPshot minisequencing extension reaction containing a pool of polymorphism-specific primers. The reaction extends the minisequencing primers by the addition of one fluorescent ddNTP, producing different products which are specific for each SNP allele. We performed the SNaPshot minisequencing reaction in a 10 μl final volume using 1 μl of treated PCR product, 5 μl of the minisequencing primer cocktail (final primer concentrations ranged between 0.02-0.2 μM for *BCL11A* and 0.05-0.3 μM for *HBS1L-MYB*, see Table 3) and 4 μl of SNaPshot® Multiplex Ready Reaction Mix (Applied Biosystems). The primer extension conditions consisted of 96°C for 10 sec, followed by 25 cycles for 10 sec at 96°C, 5 sec at 52°C; 30 sec at 60°C. Afterwards, the samples were treated by the addition of 1X Antarctic Phosphatase Buffer and 0.5 units of Antarctic phosphatase (New England Biolabs) at 37°C for 1 hour followed by enzyme inactivation at 75°C for 15 min.

Analysis of the treated minisequencing products was performed by capillary electrophoresis on an ABI 3130*xl* Genetic Analyzer (Applied Biosystems) preceded by a denaturation step. A mix of 8.5 μl of HiDi™ formamide (Applied Biosystems) and 0.5 μl of GeneScan-120 LIZ size standard (Applied Biosystems) was added to 1 μl of treated single-nucleotide extension product and denatured at 95°C for 5 min. The mixture was immediately transferred on ice for 5 min and loaded for electrophoresis onto the ABI 3130*xl* genetic analyzer (Applied Biosystems) using a 36-cm capillary, POP-7 polymer and the DS02 matrix (Applied Biosystems) for 20 minutes with 10 seconds of injection following the manufacturer’s instructions. Multiplex extension products were visualized and analyzed automatically with GeneMapper™ v4.1 Software (Applied Biosystems).

### Minisequencing genotypic profile verification

To verify the genotyping of the selected *BCL11A* and *HBS1L-MYB* polymorphisms obtained with the multiplex SNaPshot minisequencing assay we carried out Sanger DNA sequencing and/or PCR-RFLP analyses of 25 random β-thalassaemia samples. For sequencing, multiplex amplified mPCRs (A and B) products were purified using the QIAquick PCR purification kit (Qiagen). Nine cycle sequencing amplification reactions were prepared for *BCL11A* and thirteen for *HBS1L-MYB,* in each case using 20-30 ng purified mPCR product, 0.5X Big Dye Terminator v1.1 Sequencing Buffer [5X] (Applied Biosystems), 0.5X Terminator ready reaction mix v1.1 [2.5X] (Applied Biosystems), sequencing primer at a concentration of 0.5 μM (see Table 2), and double distilled water to a final volume of 10 μl. The amplification cycling profile was performed in a Veriti 96-well Thermal Cycler (Applied Biosystems) and initialized by 96°C for 1 min, followed by 25 cycles for 10 sec at 96°C, 5 sec at 50°C; 4 min at 60°C. The samples mixtures were sequenced by the ABI 3130*xl* genetic analyzer (Applied Biosystems) following the manufacturer’s instructions.

The PCR-RFLP method was applied for the detection of two SNPs at the *BCL11A* locus (rs7606173, rs6706648) and five SNPs at the *HBS1L-MYB* locus (rs28384513, rs11759553, rs9376090, rs4895440, rs4895441). Singleplex PCR reactions were performed in a final volume of 25 μl using 100 ng of genomic DNA, 1X Amplitaq Gold Buffer (Applied Biosystems), 200 mΜ of each dNTP (Invitrogen), 1.25 units of Amplitaq Gold DNA Polymerase (Applied Biosystems) and primers at 0.2 μM concentration as shown in Table 2. The thermocycling conditions were exactly the same as for the mPCRs. 8 μl of PCR product was digested with 1 μl restriction enzyme (5 units/ μl) (for *BCL11A* locus AvaII was used for rs7606173 SNP; AatII for rs6706648, and for *HBS1L-MYB* locus BstxI was used for rs28384513; RsaI: rs11759553; MspI: rs9376090; HinfI: rs4895440; and Hpy188III: rs4895441, all restriction enzymes were purchased from New England Biolabs) for 1 hour at 37°C and visualized in 2% w/v ethidium bromide-stained agarose gel to distinguish between the genotypes of each SNP allele on the basis of susceptibility to digestion by the enzymes.

## Results

### Detection of 12 *BCL11A* and 16 *HBS1L-MYB* SNPs by multiplex minisequencing assays

In order to generate the genetic profile for SNPs located in two of the most common genetic modifiers of HbF, we developed two multiplex minisequencing assays that can be applied to genotype twelve *BCL11A* and sixteen *HBS1L-MYB* polymorphisms, respectively (Table 1). Our selection of SNPs to be studied was limited to well-characterized SNPs [9-13,24-33] with a proven association with variable HbF levels in a number of different populations with HPFH, β-thalassaemia, sickle cell anaemia and heterozygotes for β-thalassaemia or HbE. 25 β-thalassaemia samples were utilized in our laboratory for the establishment of the genotyping protocol. No prior information was available regarding the *BCL11A* and *HBS1L-MYB* regions for the samples studied.

Multiplex SNaPshot minisequencing is achieved by mixing primers of different lengths with the SNaPshot mix and the multiplex PCR template. Products of this reaction differ notably in size and are produced by simultaneous multiplex single fluorescent base extension at the 3′-end of each primer [37]. Each fluorescent fragment is assigned a size based on its relative mobility with respect to the GeneScan-120LIZ size standard (15-120 nt) as it migrates through the POP-7 polymer during electrophoresis. The major and minor alleles are differentiated based on the emission of the fluorescent-dye-labelled ddNTP terminator. The analyzed fragments are represented as coloured peaks by the analysis software as follows: green for A; black: C; blue: G; and red: T [37]. In this way the two different polymorphic allele sites can easily be distinguished. Samples that are heterozygous for one interrogated SNP are expected to display two peaks, one for the major allele and one for the minor while homozygotes display one peak which corresponds either to the major or to the minor allele. The relative size of the multiplex minisequencing primers determines the order of the extension products (peaks) on the electropherogram; nevertheless, the mobility of the labeled extension products through the polymer is also influenced by the molecular mass of the label dye, so the observed product size can diverge from the actual size of the primer. Another factor is the nature of the polymer that is used: fragments smaller than 50 bp migrate with slightly different mobilities through the POP-7 polymer than expected resulting in a minor shift compared to what one would anticipate based on the relative size of the minisequencing primer (User Bulletin: Using the SNaPshot® Multiplex System with the POP-7™ Polymer on Applied Biosystems 3730/3730xl DNA Analyzers and 3130/3130xl Genetic Analyzer, Applied Biosystems 2005, P/N. 4367258 Rev. B). During design of the primers avoiding hairpins, primer self-complementarity and primer-dimers was taken into consideration because stable oligonucleotide secondary structures can affect signals.

**Figure 1:**
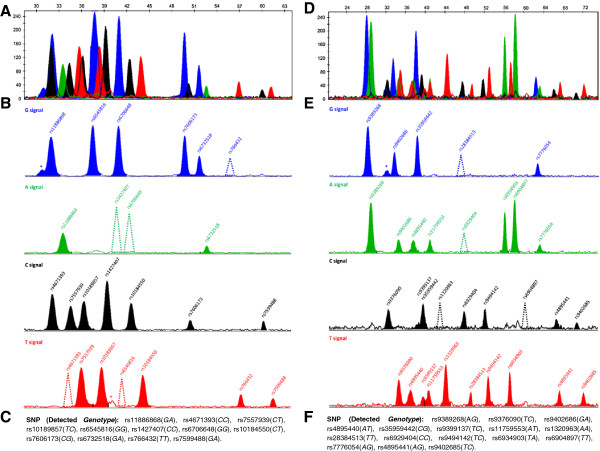
**Detection of *BCL11A *and *HBS1L-MYB *SNPs by multiplex minisequencing assays. A**. Electropherogram shows the multi detection of 12 *BCL11A* SNPs from a β-thalassaemia donor. Peaks corresponds to the fluorescence signal that is detected for each SNP. The relative fluorescence units for the detected fragments as they occurred over time are represented along y-axis and size (nt) along the x-axis. **B**. Peaks presented on 4 separate electropherograms according to their colour, which indicates the fluorescent ddNTP extension. In the cases where the sample is homozygous for a specific SNP, the alternative allele is shown with a dotted peak in order to provide an indication of its position, i.e. G for rs766432; A: rs1427407; A: rs6706648; T: rs4671393; and T: rs6545816 that do not exist in the genotypic profile of this sample. Asterisks indicate non-specific peaks; these do not interfere with the genotyping of the flanking SNPs. For SNPs detected with a reverse primer, the peak colours correspond to the complementary bases of the denoted genotype. **C**. *BCL11A* SNPs profile of the β-thalassaemia donor. **D**. Electropherogram shows the multi detection of 16 *HBS1L-MYB* SNPs from a β-thalassaemia donor. **E**. Separate coloured peaks are denoted as described in (B). Dotted peaks indicate the position of the secondary peak that represents the other allele G for rs28384513, A; rs6929404, C; rs1320963 and C; rs6904897 that do not exist in the genotypic profile of this sample. The asterisk indicates a non-specific peak which does not affect the genotyping of the flanking SNPs. **F**. *HBS1L-MYB* SNPs profile of the β-thalassaemia donor.

To examine the feasibility of the assay design and to confirm the identity of the peaks obtained, we first tested each minisequencing primer in a singleplex SnaPshot minisequencing reaction, and then in multiplex SnaPshot minisequencing reaction for each assay by using one high HbF sample and one β-thalassaemia sample with variable genotypic profiles previously determined using sequencing. During optimization of the assays, the observed size of one single-base extension product in *BCL11A* and six in *HBS1L-MYB* were affected by strong electrophoretic mobility variation. Overlaps between these and neighbouring peaks carrying identical labels resulted in poor resolution. The length of these seven primers was subsequently altered to ensure good distribution of the assay products (Table 3 contains the final minisequencing primers). Primer concentrations for use in the minisequencing assays were adjusted to generate easily readable peak heights. These were determined following several rounds of optimization. The multiplex electropherograms obtained can depict a minimum of 12 extension product peaks to a maximum of 24 peaks for the *BCL11A* assay, and 16 to 32 for the *HBS1L-MYB* assay depending on the genotype of the samples screened. The analyzed data show that in order to have sufficient electrophoretic separation between neighbouring peaks a minimum of 3 nt difference in length is required between the primers giving rise to those peaks. Taken together, our data indicate that the detectable signal for both alleles of the SNPs under investigation are obtained with high reproducibility, indicating that the 12-plex *BCL11A* and 16-plex *HBS1L-MYB* minisequencing primer set can be used for genotyping (Figure 1, https://mynotebook.labarchives.com/share/BMC%2520Genomics%2520Minisequencing%2520Data/MC4wfDIzODI4LzAvVHJlZU5vZGUvNDEyMDU0Njc2N3wwLjA). Moreover, three unexpected non-specific peaks exist in the electropherogram (two in the *BCL11A* assay and one in the *HBS1L-MYB* assay, Figure 1), though their positions do not interfere with the genotyping of the samples.

After the successful detection of all polymorphisms with these two multiplex SNaPshot minisequencing assays, 25 genomic DNA β-thalassaemia patients were tested each carrying an unknown genotypic SNPs profile. Screening the study cohort for the selected *BCL11A* SNPs by the described multiplex assay resulted in identifying all 12 heterozygote and 24 homozygote genotypes possible. For *HBS1L-MYB* SNPs screening, all 16 heterozygote and 30 homozygote genotypes were detected. The minor genotypes *AA* for rs6929404 and *GG* for rs1320963 were not observed in any of the samples.

### Validation of the multiplex minisequencing assay

To evaluate the accuracy of the assays we screened the same cohort of 25 β-thalassaemia patients as above with sequencing and/or PCR-RFLP for all 12 *BCL11A* and 16 *HBS1L-MYB* SNPs. The proportion of correctly identified SNPs (true positives) as well as the existence of any false positive or false negative peaks was examined. Our minisequencing results showed 100% agreement with the genotypes determined by sequencing and restriction fragment analysis. No false positive or false negative minisequencing results were found. These validation data prove that the new multiplex minisequencing assays developed are highly accurate and suitable for the detection of the twelve *BCL11A* and sixteen *HBS1L-MYB* polymorphisms.

## Discussion

In this study, we describe two simple multiplex assays for simultaneous detection of twelve and sixteen SNPs that are located in the *BCL11A* gene and the *HBS1L-MYB* intergenic region, respectively. These polymorphisms have been shown to have an effect on HbF levels, a major ameliorating factor of SCD and β-thalassaemia in various populations [5]. The designed assays are based on the SNaPshot minisequencing technique, which was found to be an accurate method for SNP genotyping in many biology fields including population and forensic genetics [35,38].

The assay for genotyping the selected *BCL11A* SNPs is a two-step strategy based on the multiplex amplification of nine fragments where the SNPs are located, in two separate multiplex PCR reactions, followed by one multiplex SNaPshot minisequencing reaction. Similarly, genotyping the selected SNPs in the *HBS1L-MYB* intergenic region is based on the multiplex amplification of thirteen fragments, in two separate multiplex PCR reactions, followed by one multiplex SNaPshot minisequencing reaction.

To validate the SNP genotyping results obtained by SNaPshot minisequencing we performed Sanger DNA sequencing or PCR-RFLP analyses of 25 DNA samples. The results of Sanger DNA sequencing or PCR-RFLP were always in agreement with those of the SNaPshot minisequencing.

Using the SNaPshot minisequencing technique we were able to determine the genotypes of the samples based on the position and the fluorescent colour of the peaks on the electropherograms generated. The minor shifts observed in the position of the peaks compared to the expected size may be due to the different molecular weights of each of the four fluorescent ddNTPs which can be incorporated at the end of each primer and to the POP-7 polymer used. These shifts are consistent for all samples that were tested and did not interfere with the analysis of the polymorphisms. Neutral tails incorporated at the ends of the minisequencing primers ensured sufficient spacing of the peaks and accommodated any unexpected small shifts in peak positions.

**Figure 2:**
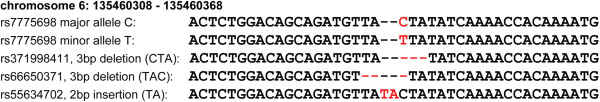
**Sequence variations close to SNP rs7775698.** Nucleotide sequences between chromosome 6: 135460308 to 135460368 bp. The sequences shown are: the rs7775698 major allele C, the rs7775698 minor allele T, the rs371998411 (CTA) 3 bp deletion, the rs66650371 (TAC) 3 bp deletion and the rs55634702 (TA) 2 bp insertion. In red is indicated the sequence variation.

A crucial step for the success of the minisequencing assay is the correct design of the multiplex minisequencing primers. Given that the 3′ end of the minisequencing primer must be immediately adjacent to the SNP of interest, there is little flexibility in the positioning of the primer, although parameters like the length of the primer and its orientation are more amenable to manipulation. In deciding the orientation of the primers, it is necessary to take into consideration the presence of any additional sequence variations flanking the SNP of interest as these may cause problems in the annealing and extension of the primer [39,40]. It is for this precise reason that we did not include SNP rs7775698 in the assay for genotyping the *HBS1L-MYB* intergenic region even though this SNP is known to be associated with elevated levels of HbF [31,32]. Specifically, 3 more SNPs overlap with or are immediately adjacent to SNP rs7775698. These are rs371998411 and rs66650371, which are 3 bp deletions, and rs55634702, which is a 2 bp insertion (Figure 2). Consequently, designing a single minisequencing primer either upstream or downstream of the SNP rs7775698 is not sufficient to genotype the SNPs at this position. Sanger DNA sequencing is one of the techniques that can be used to resolve this issue.

Based on our observations that the multiplex PCR reactions for both the *BCL11A* and *HBS1L-MYB* assays were well standardized and that no amplification problems were experienced for any of the samples that we genotyped, we concluded that it is possible to reduce the time necessary for the completion of the whole assay by omitting the agarose gel electrophoresis step for the detection and analysis of the initial multiplex PCR products. Moreover, the assay can be easily customized for the analysis of only a subset of the SNPs or the addition of more SNPs without major changes in the conditions for either assay.

The advantage of the minisequencing technique as a means of screening for polymorphisms is that several selected polymorphisms can be detected simultaneously in a single reaction, with the results presented in a single electropherogram. The technique can be easily performed in any laboratory that is equipped with a thermocycler and a DNA sequencer. The SNaPshot minisequencing technique is highly accurate and sensitive and is suitable for the genotyping of large numbers of clinical samples relatively quickly. The possibility of using this technique for simultaneous identification of several polymorphisms at distant genetic locations in a single reaction makes it cost and time efficient compared to other commonly used genotyping techniques such as PCR-RFLP and Sanger DNA sequencing. Although PCR-RFLP is a simple technique, its use for genotyping the large numbers of SNPs selected for the two loci under investigation is time consuming and impractical because several PCR reactions must be carried out per locus. More importantly, PCR-RFLP is not applicable for all SNPs under investigation because of the absence of informative restriction enzyme recognition sites for all SNPs or the presence of additional SNPs within the recognition site. Similarly, Sanger DNA sequencing for the selected SNPs in our assays requires carrying out nine sequencing reactions for the *BCL11A* SNPs and thirteen sequencing reactions for the *HBS1L-MYB* SNPs compared to a single SNaPshot minisequencing reaction. Although it is possible to screen large scale sequences or SNPs with next generation sequencing and arrays, respectively, these high-throughput techniques are expensive and require specialized equipment which is not commonly available in most molecular biology laboratories.

In addition, the two described multiplex minisequencing assays can be combined with multiplex ligation-dependent probe amplification (MLPA) technique or Gap-PCR for rapid detection of any deletions or duplications of one or more sequences in or near the α–globin genes [41-43], since this is an important modifier of the severity of SCD and β–thalassaemia [44].

## Conclusion

The primary goal of this study was to develop a simple and rapid method to genotype SNPs in the *BCL11A* gene and in the *HBS1L-MYB* intergenic region that were shown in various studies to be associated with variable HbF levels in several populations. Multiplex SNaPshot minisequencing, a powerful method that can be used for analyzing SNPs distantly located to each other in a single reaction was chosen for this purpose. Taking the advantage of this method, two multiplex SNaPshot minisequencing assays were developed for simultaneous genotyping of twelve SNPs in the *BCL11A* gene and sixteen SNPs in the *HBS1L-MYB* intergenic region. The method was applied to screen 25 different samples and the results obtained were verified by DNA sequencing and PCR-RFLP. Concluding, the assays developed can be successfully applied for accurate, time and cost efficient simultaneous genotyping of SNPs associated with HbF levels in the *BCL11A* gene and in the *HBS1L-MYB* intergenic region in HPFH, β-thalassaemia, SCD or other hemoglobinopathy populations.

## Competing interests

The authors declare that they have no competing interests.

## Authors’ contributions

MK introduced the concept of the genotyping of *BCL11A* and *HBS1L-MYB* SNPs with the two SNaPshot Minisequencing Assays and provided advice on methodology development. PF designed the experiments, aided in the implementation of the experiments and drafted the manuscript. IK developed the laboratory protocols for the two SNaPshot Minisequencing assays to the level of standard screening procedure, performed the analysis of the data and drafted the manuscript. MP provided advice on methodology development and on manuscript writing. All authors read and approved the final manuscript.
